# Root canal morphology and variations in mandibular second molars: an in vivo cone-beam computed tomography analysis

**DOI:** 10.1186/s12903-021-01787-7

**Published:** 2021-09-01

**Authors:** Francisco Gomez, Gisbeli Brea, Jose Francisco Gomez-Sosa

**Affiliations:** Postgraduate Department of Endodontics, Universidad Central de Venezuela, Caracas, Venezuela

**Keywords:** Mandibular second molar, Anatomical variation, C-shaped canals, Radix Paramolaris, CBCT

## Abstract

**Background:**

The purpose of this study was to determine the anatomical variations of the root canal system of mandibular second molars using cone-beam computed tomography (CBCT).

**Methods:**

190 mandibular second molars cone-beam computed tomography images were reviewed. The evaluation was performed by a radiologist with endodontic experience and two endodontists trained with CBCT technology. Tooth position, number of root and root canals, C-shaped root canal system configuration, presence of extra root (radix), and radicular grooves were assessed. Data was statistically analyzed using The Chi-square test (α = 0,05) to determine any significant difference between gender and the total number of root and root canals, and any significant difference between gender and root canal anatomical variation.

**Results:**

Overall, 85.5% showed two separated roots, 12.1% a single root, 2.6% three roots or radix. 87.7% showed three root canals, 12.1% two root canals, 2.6% four root canals, and 1.6% a single root canal. 10% showed a single foramen, 75.3% two foramina, 13.6% three foramina and 1% showed four foramina.19.5% showed C-shaped anatomical variation, 51.4% in male patients, 48.6% in female patients. According to Fan classification: C1 13.6% in cervical third, C2 10% in the middle third, C3 17.3% in middle third, 15.5% in apical third, and C4 12.7% in the apical third. Root canals number in these samples were 5.4% a single canal, 21.6% two canals, 70.3% three canals, and 2.7% four canals. The root showed 46% with one foramen, 46% two foramina, and 8% three foramina. Radicular grooves 83.3% were found in the lingual area and 16.2% towards the buccal area.

**Conclusions:**

The most prevalent anatomic presentation of the evaluated sample was a mandibular second molars with two roots, three root canals, and two apical foramina. Their variation was C-shaped root canals and Radix Paramolaris.

## Background

Knowledge of the morphology of the root canal system is essential for the correct diagnosis of anatomical variation before starting the endodontic therapy [1]. Mandibular second molars usually have two roots with three root canals, two in the mesial root and one in the distal root; however, these teeth can present severe anatomical variations, such as the presence of three canals in the mesial root, two canals in the distal root, or supernumerary roots [2].

A C-shaped configuration is within the anatomical variants that can be found on second molars, this was first described in 1979, by Cooke and Cox [3] as a consequence of an alteration in root development due to the lack of fusion of the Hertwig's root epithelial sheath of the vestibular or lingual side [4]. The C-shaped anatomical configuration can be as a single ribbon or an isthmus connecting individual root canals [5, 6]. Some studies reported C-shaped root canal prevalence between 2.7% to 8%, more frequent in the Asian population or white race. This variation seems to be associated with their ethnic [4, 6]. Seo and Park observed that these root canals have a high possibility of splitting into two or three canals in the apical third, so this particular canal anatomy is not predictable based only on the shape of the pulp chamber [7].

On the other hand, mandibular second molars can have root number variations with a supernumerary root. When this additional root is located in a disto-lingual side it is called radix entomolaris, and if is located on the mesio-buccal side it is called radix paramolaris [8]

For the analysis of mandibular second molars with a C-shaped configuration, different study techniques of their characteristics have been implemented; including cross-sections and microcomputed tomography in teeth that have been extracted [9]. In endodontics, the best method for an accurate determination of morphology is the CBCT. Which allows the endodontist or clinician to make better diagnoses and decision-making before the start of treatment. This accurate tool offers three planes of analysis of the teeth with more reliable images, compared to a periapical or panoramic radiograph [10, 11].

CBCT evaluations have made it possible to carry out studies of mandibular second molars in populations such as China, Indian, Korea, Brazilian, Portuguese and Israeli, allowing them to know the characteristics of anatomical variations, and the percentage of appearance of the population studied. The present study aimed to determine the anatomical variations of the root canal system of mandibular second molars using cone-beam computed tomography (CBCT).

## Methods

### Sample selection

This study was approved by The Ethics Committee of Dentistry School to the Universidad Central de Venezuela. The CBCT images of mandibular second molars were acquired from patients who required a preoperative assessment as part of their dental examination, diagnosis, and treatment planning from X-ray institute in Caracas, Venezuela between January 2014 and December 2017.

A convenience sample of 190 mandibular second molars from a total of 967 CBCTs of the period above mentioned, corresponding to 161 Venezuelan patients were selected according to the following criteria: the presence of CBCT images of mandibular second molars with complete root formation, absence of previous root canal treatment, and absence of root resorption or periapical pathosis. CBCT mandibular full arches, with the presence of both second molars, were taken as two samples.

### Image acquisition

The CBCT images were obtained using Kodak 9000 3D unit (Carestream Dental, Atlanta, GA, USA); at 60–90 kV and 2–15 mA with an exposure time of 2–6 s. The voxel size of the images was 76 × 76x76, and the slice thickness was 200 um with 16 bits Grayscale. An experienced radiologist performed the acquisition process according to the manufacturer's recommended protocol with the minimum exposure necessary for adequate image quality. According to human ethics procedures, all methods were carried out following relevant guidelines and regulations [12].

### Image evaluation

All the images from 190 mandibular second molars were evaluated with a 3D Imaging Software 3.3.9.0 (Carestream Dental LLC, Atlanta, GA. USA) and a Dell Inspiron 15 5000 Laptop (Intel® Core™ i5-1135G7, Processor 8 MB Cache, up to 4.2 GHz. Windows 10 Pro 64-bit English) according to Brea et al. methods [12].

Two endodontists independently evaluated the images twice, with a week interval between the assessments. If there were disagreements between them, a radiologist with endodontic experience was asked to perform a third evaluation and then reach a final consensus. All the evaluators were calibrated by analyzing 20 random cases of mandibular molars based on the same criteria and variants. The Cohen´s Kappa was used to analyze the presence of anatomical variation and variation type (qualitative variable), and the intraclass correlation coefficient (ICC) was used to analyze the roots and root canals number (quantitative variable), according to Brea et al. methods [12].

Results of the first analysis showed high values of agreement with the statistical methods applied: Presence of Anatomical Variations: 0.91 Cohen's Kappa, Variation Type: 0.92 Cohen's Kappa, Roots Number: 0.89 ICC, and root canals number: 0.91 ICC. Results of the second calibration between specialists showed the same high values ​​ of agreement: Presence of Anatomical Variations: 0.90 Cohen's Kappa, Variation Type: 0.94 Cohen's Kappa, Roots number: 0.91 ICC, and root canals number: 0.89 ICC.

Then the following information of 190 mandibular second molars were recorded:

- Tooth position: right or left mandibular second molars.
- The number of roots, root canals, and apical foramina.
- Presence of radix, and their position.
- Root canal configuration according to Vertucci´s classification.
- C-shaped root canal system configuration.
- C-shaped classification over the cervical, middle and apical third, according to Fan et al. criteria [5]:
- (C1): the shape was a continuous “C” with no separation or division;
- (C2): the canal shape resembled a semicolon resulting from discontinuation in the "C" outline.
- (C3): two or three separate round, oval, or flat canals.
- (C4): only one round, oval, or flat canal in that cross-section.
- (C5): no presence of a canal.
- Radicular grooves: vestibular or lingual area.

The total number of roots and root canals, incidence of root canal anatomical variation, and the correlations between occurrences in males and females were analyzed. Data were statistically analyzed using the Chi-square test with SPSS 21.0 for Windows (SPSS Inc, Chicago, IL), with significance set at *p* < 0.05.

## Results

**Fig. 1:**
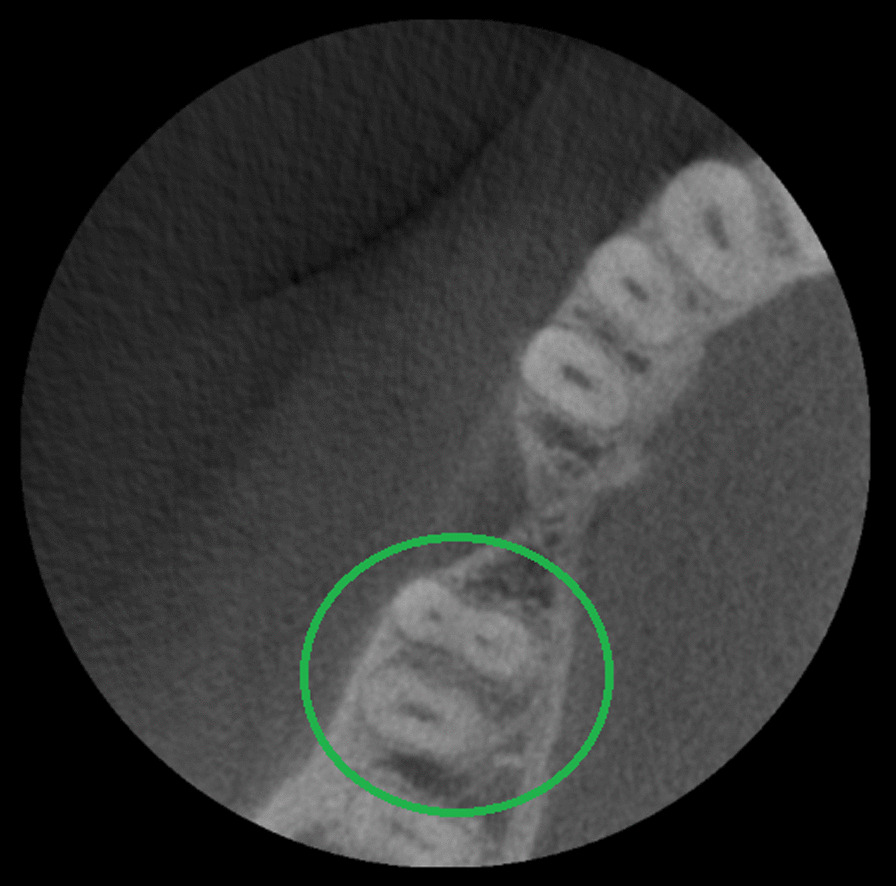
Axial view from CBCT scan showing mandibular second molar with two roots

**Fig. 2:**
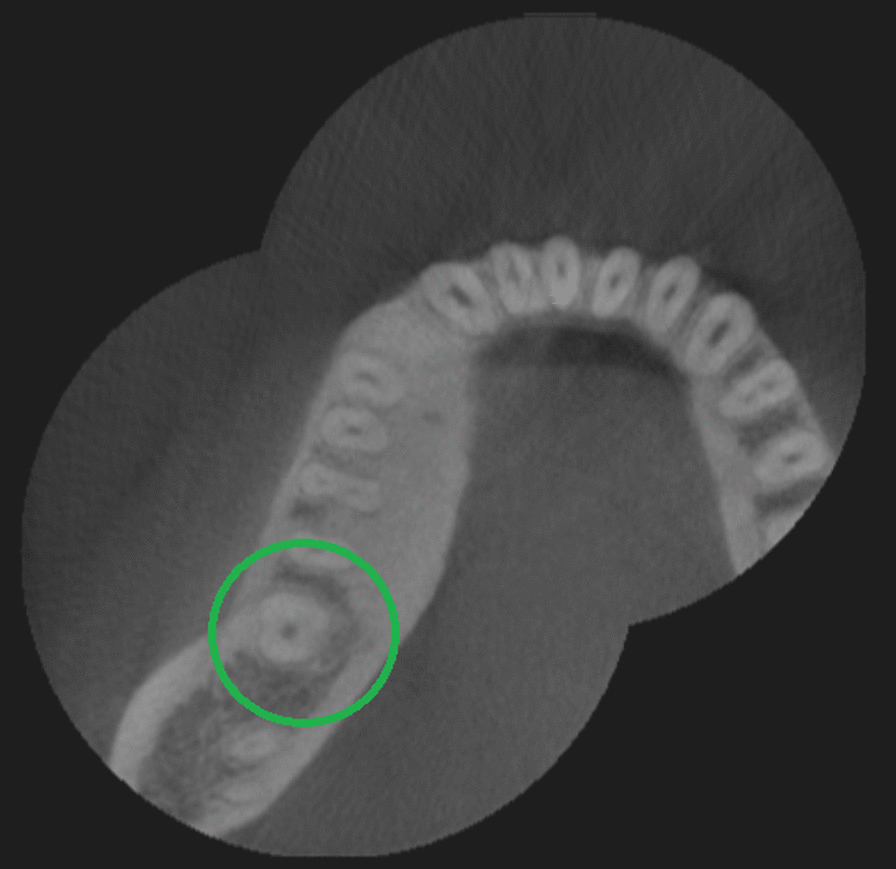
Axial view from CBCT scan showing mandibular second molar with one root

**Fig. 3:**
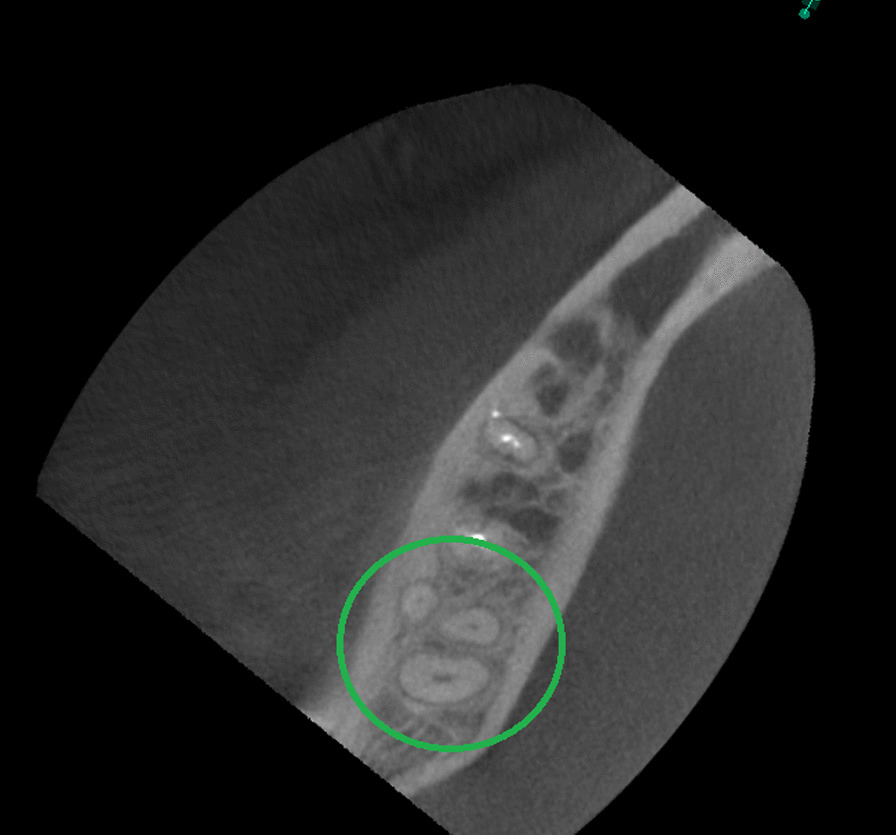
Axial view from CBCT scan showing mandibular second molar with three roots (Radix Paramolaris)

One hundred ninety mandibular second molars (100%) were evaluated; 42.6% were male patients and 57.4% female patients. In regards of tooth position, 51.6% were mandibular left second molars and 48.4% mandibular right second molars. Their root numbers showed two separated roots in 85.3% cases (Fig. 1), single-rooted in 12.1% cases (Fig. 2), and three roots or radix Paramolaris in 2.6% cases (Fig. 3). 87.7% mandibular second molars showed three root canals, 12.1% two root canals, 2.6% four root canals, and 1.6% a single root canal. 10% cases showed a single foramen, 75.3% two foramina, 13.6% three foramina and only 1% showed four foramina (Table 1).

**Table 1:** Number of roots, root canals and c-shaped anatomical variation in mandibular second molars and gender

Roots number	Total (%)	Gender	
		Males (%)	Females (%)
T o t a l	190 (100,0)	81 (42,6)	109 (57,4)
One	23 (12,1)	12 (52,2)	11 (47,8)
Two	162 (85,3)	64 (39,5)	98 (60,5)
Three (Radix)	5 (2,6)	5 (100,0)	0
Root canals number	Gender		
One	3 (1,6)	0	3 (100,0)
Two	23 (12,1)	13 (56,5)	10 (43,5)
Three	159 (83,7)	64 (40,2)	95 (59,8)
Four	5 (2,6)	4 (80,0)	1 (20,0)
C-shaped anatomical variation	Gender		
Total	37 (19.5)	19 (51.4)	18 (48.6)

According to Vertucci´s classification the most frequent mesial root canals configuration in mandibular second molar was type II (74.3%), type IV (12.1%) type I (6.7%), and Type III (6%). The sample evaluated in distal root canals configuration showed 97.2% type I, 0.51% type V and 1.02% type II (Table 2).

**Table 2:** Root canal configuration of second molars according to Vertucci´s classification

Vertucci´s	I	II	III	IV	V	VI	VII
Mesial root	6.70%	74.30%	6%	12.10%	-	-	-
Distal root	97.20%	1.35%	-	-	0.60%	-	-

### C-shaped configuration mandibular second molars

**Fig. 4:**
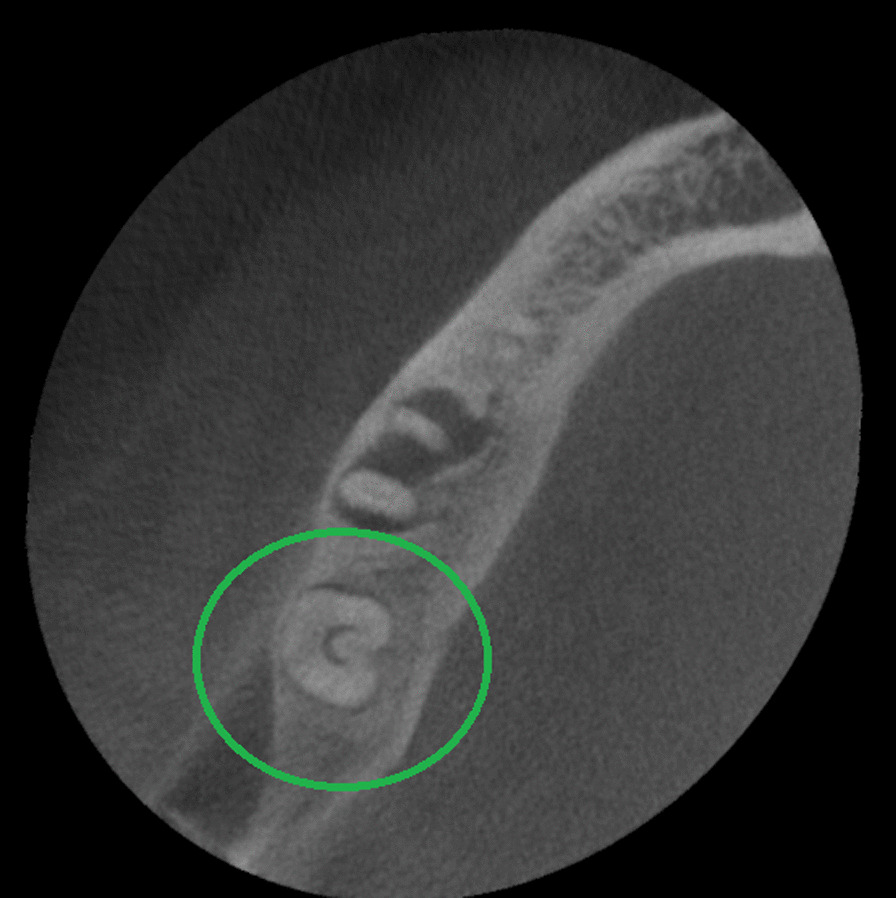
Axial view from CBCT scan showing mandibular second molar with C-shaped root canal system configuration C1 according to Fan et al. [4]

**Fig. 5:**
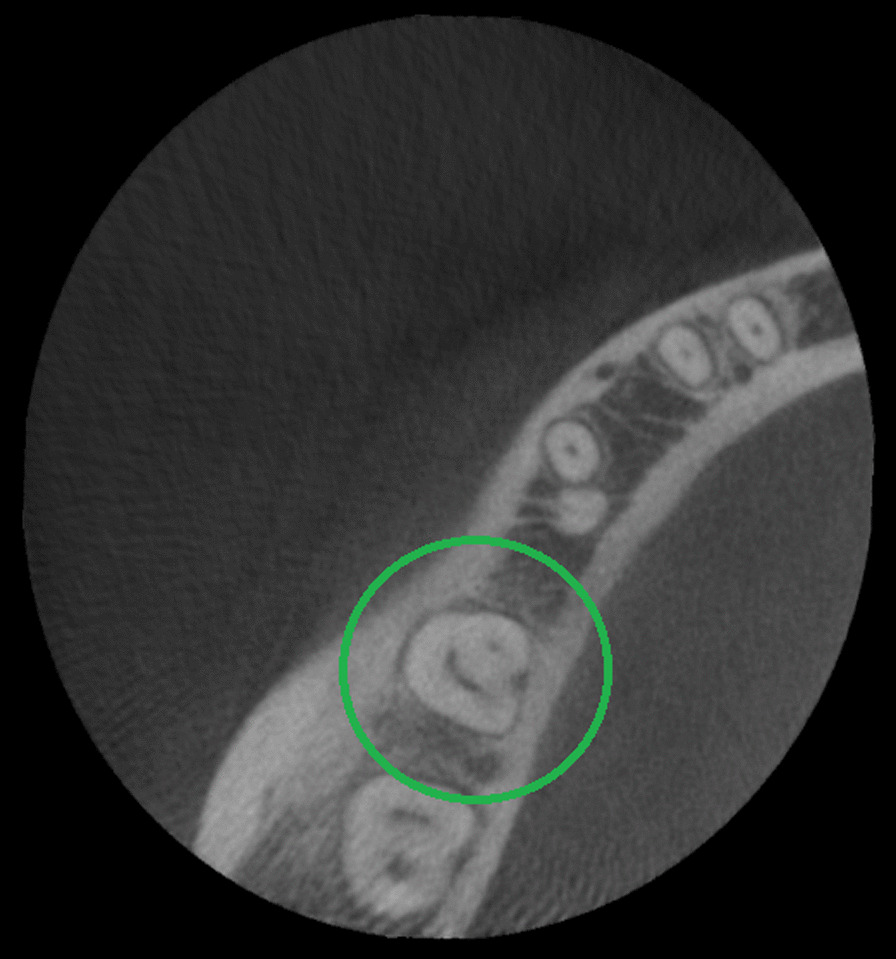
Axial view from CBCT scan showing mandibular second molar with C-shaped root canal system configuration C2 according to Fan et al. [4]

**Fig. 6:**
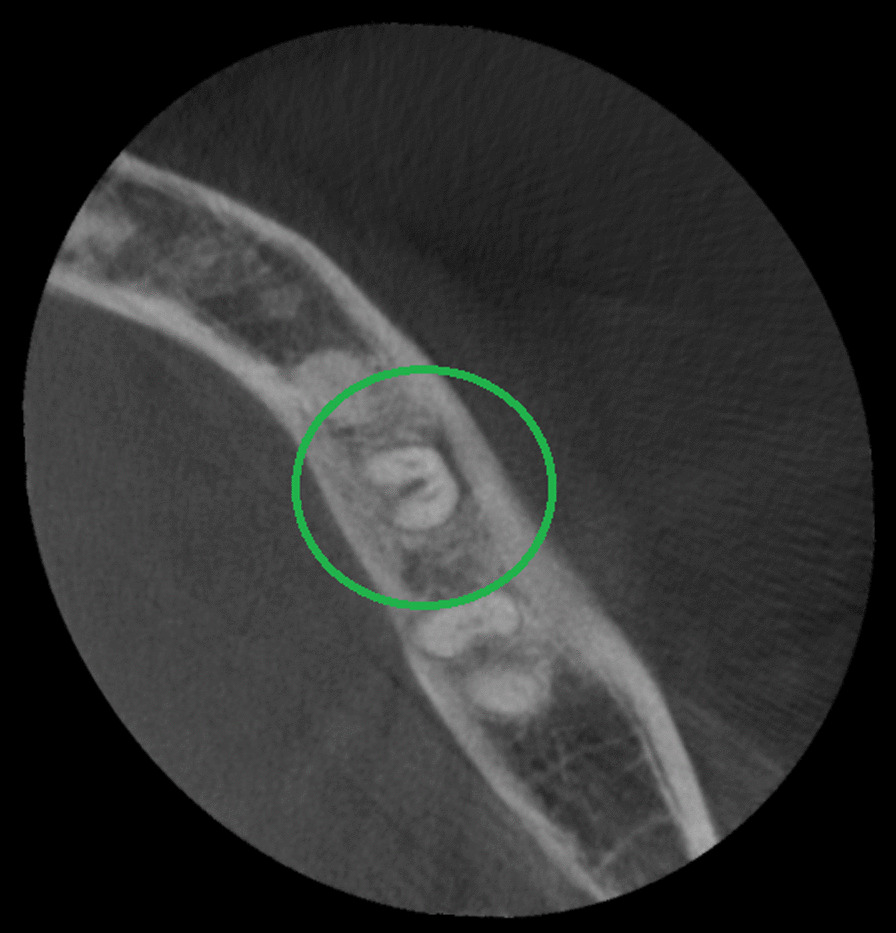
Axial view from CBCT scan showing mandibular second molar with C-shaped root canal system configuration C3 according to Fan et al. [4]

**Fig. 7:**
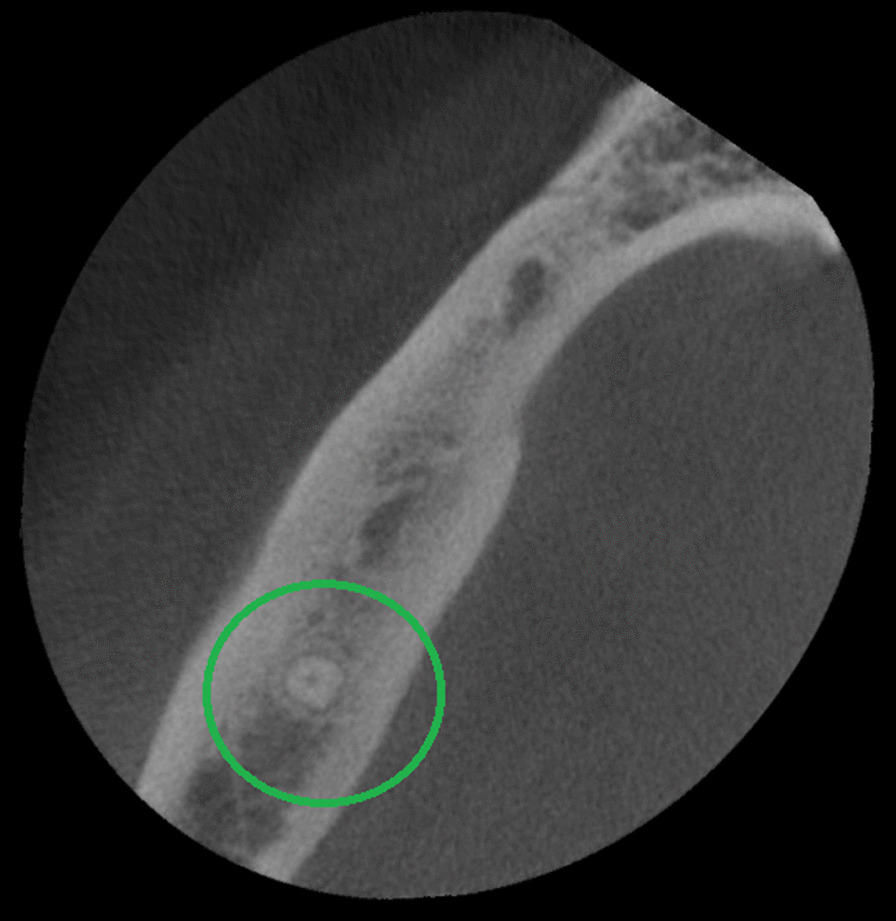
Axial view from CBCT scan showing mandibular second molar with C-shaped root canal system configuration C4 according to Fan et al. [4]

The presence of a C-shaped root canal system was observed in 37 mandibular second molars representing 19.5% of the total samples (Table 1). 19 cases (51.4%) for male patients and 18 (48.6%) for female patients (Tables 3 and 4). According to Fan et al. [5] classification, these mandibular second molars were identified as C1 13.6% in cervical third (Fig. 4), C2 10% in the middle third (Fig. 5), C3 17.3% in middle third, 15.5% in apical third (Fig. 6), and C4 12.7% in the apical third (Fig. 7) detailed information by gender see Tables 3 and 4. The root canals number in these samples were 5.4% a single canal, 21.6% two canals, 70.3% three canals, and 2.7% four canals. The root showed 46% with one foramen, 46% two foramina, and 8% three foramina. Radicular grooves in the C-shaped second molars under study were found as follows: 83.3% in the lingual area and 16.2% towards the buccal area.

**Table 3:** Cross-sectional c-shaped mandibular second molars at different root levels, according to Fan et. Al [5] in male patients

Root level	C1%	C2%	C3%	C4%
Cervical	52.63	26.31	21.05	0
Middle	26.31	36.84	42.10	0
apical	10.52	5.2	52.63	31.57

**Table 4:** Cross-sectional c-shaped mandibular second molars at different root levels, according to Fan et. al [5] in female patients

Root level	C1%	C2%	C3%	C4%
Cervical	27.77	22.22	27.77	22.22
Middle	5.55	27.77	55.55	11.11
apical	5.55	5.55	44.44	44.44

According to gender and root canal morphology, male patients showed 33.68% (n = 64) mandibular second molar with two roots and 2.63% (n = 5) with three roots. Female patients showed 51.57% (n = 98) mandibular second molar with two roots and any sample with three roots. No significant difference was found between gender and root numbers (*p* > 0.05). Nor in the prevalence of C-shaped configuration and gender (*p* > 0.05).

## Discussion

This study highlights the importance of anatomy knowledge of the root canal system, for the clinician. The correct diagnosis of an anatomical variation, such as an accessory root or presence of a C-shaped canal, before starting an endodontic therapy facilitates its development. Also, knowledge of the frequency and location of accessory roots (radix), could guide to modified access to the pulp chamber. Variations in mandibular second molars show large anastomoses, lateral canals, and apical deltas, hindering a correct cleaning and shaping of root canals, so the professional must also modify some techniques in the endodontic therapy to avoid procedural mistakes to obtain a correct obturation and treatment success [1, 7, 8, 13, 14].

CBCT provides an excellent, non-destructive, noninvasive imaging option with the potential to detect most anatomic variations while creating an accurate representation of the external and internal dental anatomy. The quality of CBCT is sufficiently high to visualize root canal morphology before an endodontic treatment at low radiation and dosimetry [15].

Venezuela is a variety of ethnic countries, to our knowledge, it does not have statistical studies about anatomical variation in mandibular second molars. For this reason, the present study used a database from patients of a Venezuelan imaging diagnostic center to analyze their possible variations and characteristics.

Base on the CBCT analysis of 190 mandibular second molar, the results show that 85.3% have two roots located in mesiodistal direction, 12.1% a single root, and 2.6% three roots. These findings match with the studies by Pawar et al. in 2015, [14] which obtained a prevalence of 79.35% of second molars with two roots in an Indian population, and Von Zuben et al. in 2017 [16] which reported 83% incidence of second molars with two roots in different countries populations. On the other hand, Kim [17] evaluated separate roots in a Korean population, their results showed 57.4% of prevalence, lower than the present study, which may be due to differences in races or number of samples.

The 83.7% of cases showed three root canals and 75.3% two foramina, matches with Neelakantan [18], Pawar [14], and Martins [10, 11] studies. According to Vertucci´s classification the most frequent configuration mesial root canals in mandibular second molar was type II (74.3%), different with Pawar [14] and Neelakantan [18] studies, who obtained type IV as higher prevalence. In distal root canals the type I was the most common, like Kim [17], Perez-Heredia [21] and Pawar [14] studies. The Vertucci´s configuration in radix paramolaris was 100% type I in mesial and distal root canals of the samples.

The C-shaped root canal system was represented 19.5%. Similar with Mexico 14.2% [16] and Indian population study 13.12% [14]. And different with China 44% [16], Korean 40% [17] and Brazil 6.8% [16] population. The discrepancy may be due to differences in races, the number of samples, analysis technique, and application of statistical parameters.

According to Fan et al. [5] classification, the most common C-shaped configuration was C1 (13.6%) in the coronal third, matching with Kim [17] study and in discrepancy with Pawar [14] who obtained higher C2 prevalence. C3 (17.3%) in the middle third, opposite with Pawar [14] who obtained more C2. C4 (12.7%) in apical third, opposite to Kim [17] and Pawar [14] who found more C3.

Radicular grooves were located in the lingual surface in 83.3% of the cases, similar to Kim [17] investigation, and 16.2% towards the buccal area, opposite to a 1% reported by Kim [17].

According to the gender and presence of C-shaped configuration in the 100% of the C-shaped samples studied, male patients showed 51.4%, and female patients 48.6%. with no significant statistical difference as Zheng [3] study. However, females showed a higher prevalence of C-shape mandibular second molars in most other investigations [16, 19].

The present study showed 2.6% of Radix Paramolaris, similar to Shemesh [20] investigation. This is a rare anatomical variation, with a significant difference between genders. The characteristic observed was the same direction, length, and curvature as the main root. All the extra roots were found in male patients. On the other hand, a Korean population study [17] showed 0.72% of extra roots in mandibular second molars, and in a Spanish population study [21] this variation was not found.

The importance of proper diagnoses and analysis of cases with anatomical variation implies the application of rigorous criteria in clinical action. In cases of mandibular second molars with C-shaped anatomical variation, use magnification and adequate preparation to avoid excessive removal in dentinal thinness walls near to invagination zone, copious irrigation with sodium hypochlorite, EDTA, and ultrasonic activation is recommended, as well as obturation with thermoplastic gutta-percha to guarantees better results of the endodontic therapy [2]

## Conclusion

Mandibular second molars of the Venezuelan population studied mostly showed two roots located in the mesiodistal direction, with three root canals and two apical foramina. Regarding C-shaped roots canals the prevalence was high in relation to other studies carried out in other regions.

## Data Availability

The datasets used and/or analyzed during the current study are available from the corresponding author on reasonable request.

## Abbreviation

CBCT: Cone-beam computed tomography
